# Disadvantage Indices Deployed to Promote Equitable Allocation of COVID-19 Vaccines in the US: A Scoping Review of Differences and Similarities in Design

**DOI:** 10.1001/jamahealthforum.2021.4501

**Published:** 2022-01-21

**Authors:** Tuhina Srivastava, Harald Schmidt, Emily Sadecki, Melanie L. Kornides

**Affiliations:** 1 Department of Biostatistics, Epidemiology and Informatics, Perelman School of Medicine, University of Pennsylvania, Philadelphia; 2 Department of Medical Ethics and Health Policy, Perelman School of Medicine, University of Pennsylvania, Philadelphia; 3 Department of Family and Community Health, School of Nursing, University of Pennsylvania, Philadelphia

## Abstract

This scoping review identifies the construction and defined purpose of disadvantage indices deployed during the initial COVID-19 vaccine rollout.

## Introduction

To promote equitable allocation of COVID-19 vaccines, expert guidance proposed to incorporate statistical, place-based measures of disadvantage (“disadvantage indices”) into allocation frameworks^1^ by allocating larger shares of vaccines to disadvantaged communities, planning dispensing site locations, and/or through targeted community outreach.^1,2^ Disadvantage indices combine metrics such as income, housing quality, and education, enabling ranking at a particular geographic unit, such as the census tract. By the end of March 2021, the majority of US states (n = 37) used various disadvantage indices to inform COVID-19 vaccine allocation and planning, but these indices differ in design.^2^ Our objective was to review the construction and defined purpose of indices deployed during the initial COVID-19 vaccine rollout.

## Methods

All Centers for Disease Control and Prevention jurisdictional health departments’ websites were queried in a structured search from November 2020 to March 2021 to retrieve COVID-19 vaccination allocation plans, as documented in a previously published review.^2^ Institutional review board review was not required owing to the use of publicly available index data sets that did not contain human participants’ information. Two authors (T.S. and E.S.) extracted index design methods and data from source websites or published methods articles, and 2 authors (H.S. and M.L.K.) reviewed extracted data (eMethods in the Supplement). Indices without publicly available methods and data were excluded. Categories used for index characterization are shown in the Table.

**Table.** Summary of Characteristics of Disadvantage Indices

Characteristic	Area Deprivation Index (ADI)	COVID-19 Community Vulnerability Index (CCVI)	California Healthy Places Index (HPI)	Social Vulnerability Index (SVI)
Year developed/current data year	2013/2019	2020/Multiple	2015/2018	2011/2018
Frequency of update	Every 5 y based on US Census Bureau American Community Survey 5-y estimates	Unclear	Unclear	Every 2 y based on US Census Bureau American Community Survey data releases
Purpose	To share measures of neighborhood disadvantage with the public for use in research, program planning, and policy development	To assess community resilience to the COVID-19 pandemic	To assist Californians in exploring local factors that predict life expectancy and comparing community conditions across the state	To identify communities that need support throughout natural disasters or human-made hazardous events
Data sources	American Community Survey, US Census Bureau	Numerous	Numerous	American Community Survey, US Census Bureau
Geographic unit reported	Census block group^a^	Census tract^b^ County State	Census tract^b^ County As well as: City/census-designated place Census zip code tabulation area Elementary school districts Medical service study areas Federal congressional districts State assembly districts State senate districts Census core-based statistical areas Metropolitan planning organizations	Census tract^b^ County
No. of variables	17	40	24	15
Index-defined construct domains	Income Education Employment Housing quality	SES Minority status and language Housing type, transportation, household, composition, and disability Epidemiological factors Health care system factors High-risk environments Population density	Economy Education Health care access Housing Neighborhoods Clean environment Transportation Social environment	SES Household composition and disability Minority status and language Housing type and transportation
Weighting of variables/domains	Weighted based on factor score coefficients for individual variables (need to integrate all variables/domains)	Variables weighted equally (need to integrate all variables/domains)	Weighted sum of regression domain scores to maximize the correlation of life expectancy at birth with the overall HPI score (need to integrate all domains)	Variables weighted equally (permissible to omit variables)
Ranking level	National State	National State	State (California)	National State
Index construction	Geographic areas are ranked by national percentile rankings: ranks 1-100 at the block group level and in deciles from 1-10 for each state. A ranking of 1 indicates the lowest level of disadvantage within the nation and a ranking of 100 indicates the highest level of disadvantage.	Each geographic area is ranked relative to one another on a 0-1 scale, with 0 being least vulnerable and 1 being the most vulnerable.	Geographic areas are assigned a percentile rank: ranks range from 0-100 with those closer to 100 indicating healthier community conditions.	Geographic areas are ranked based on percentile ranks: ranks from 0-1, with higher values indicating greater vulnerability.

Abbreviation: SES, socioeconomic status.

^a^ Census block groups contain approximately 600 to 3000 people.

^b^ Census tracts contain approximately 1200 to 8000 people.

## Results

We identified 8 indices,^2^ of which 4 had publicly available methods and data and were included in this review: Area Deprivation Index (ADI),^3^ COVID-19 Community Vulnerability Index (CCVI),^4^ Healthy Places Index (HPI),^5^ and Social Vulnerability Index (SVI)^6^ (Table, Figure). The ADI is intended to be a general planning and health policy tool.^3^ The CCVI focuses specifically on COVID-19.^4^ The HPI measures how various social determinants of health influence life expectancy at birth in California.^5^ The SVI, the most widely used index, centers on responses to natural disasters.^2,6^ Indices capture communities at differing geographic levels: block group (600-3000 people), census tract (1200-8000 people), zip code, and county. The HPI also ranks by numerous administrative areas (eg, school districts). The ADI, CCVI, and SVI report national and state-level rankings. The HPI reports rankings specific to California (Table). Indices range from 15 (SVI) to 40 variables (CCVI). We grouped 78 total variables that indices use under 9 overarching domains based on index-defined domains: population demographics, poverty, education and employment, racial and ethnic minority populations, housing and transportation, high-risk transmission environments, health, health care system, and environmental and neighborhood. Indices largely source variables from the American Community Survey.

**Figure.**
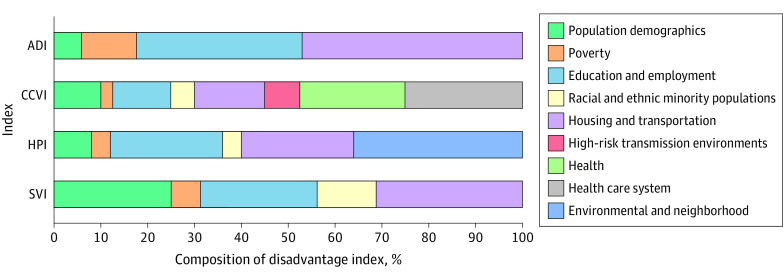
Percentage Composition of Each Disadvantage Index by Domain These 9 domains were derived from the index-defined domains in the Table. Most index-defined domain names were used or matched across indices, which had slight variations in domain naming. “Epidemiological Factors” were renamed as “Health.” All different “environmental” or “neighborhood”-specific factors were grouped under “Environmental and Neighborhood.” The colored bars indicate percentage composition of each index by the number of variables in author-constructed domains. Because the SVI and CCVI weight all variables equally, the percentages shown are representative of true domain share in index construction based on the number of variables included for those indices. ADI indicates Area Deprivation Index; CCVI, COVID-19 Community Vulnerability Index; HPI, Healthy Places Index; SVI, Social Vulnerability Index.

## Discussion

While all indices were used to promote equitable vaccine allocation, we found similarities and differences in index construction across geographic reporting units, number of variables, and weighting strategies. The ADI reports the most granular geographic units (block groups), compared with the CCVI and SVI (census tracts). Larger geographic units may mask heterogeneities in “disadvantage” in population-dense settings, such as larger cities, and may lead to underestimation of disadvantage. However, because most data are available at the census tract, one can favor pragmatically trading off accuracy for comprehensiveness of data. Additional variation is found in indices’ variable weighting, for which some indices rely on factor score coefficients (ADI) while others have fixed weights for each domain, resulting in differences even among indices with similar variables, affecting the broader concept of “disadvantage” that is captured. While this study does not quantify allocation trade-offs, it provides an important perspective on considering indices’ role and what would have happened absent their use.

The uptake of disadvantage indices to promote social justice in the initial allocation of COVID-19 vaccines was unprecedented, rapid, and widespread.^2^ It continues to be relevant during vaccination of children aged 5 to 11 years, where using indices within a tool such as the Vaccine Equity Planner (https://vaccineplanner.org) can help identify so-called vaccine deserts, as well as for prioritizing outreach and vaccination site planning for boosters and initial vaccinations as the Omicron variant amplifies the fourth COVID-19 wave. Likewise, indices hold promise for promoting equity in the allocation plans for recently approved pharmaceutical treatments. All indices used appear to be associated with benefiting vulnerable communities compared with not using an index, but future research should identify the advantages and disadvantages associated with the use of one index vs another for specific purposes.^2^

## Notes

Supplement. **eMethods.** Grouping of disadvantage index variables and domains under analytic meta-domains.
