# Supplementary material for: Disadvantage Indices Deployed to Promote Equitable Allocation of COVID-19 Vaccines in the US: A Scoping Review of Differences and Similarities in Design
Source: JAMA Health Forum. 2022 Jan 21;3(1):e214501. doi: 10.1001/jamahealthforum.2021.4501 (PMC8903102; doi:10.1001/jamahealthforum.2021.4501)
Supplement: Supplement. — eMethods. Grouping of disadvantage index variables and domains under analytic meta-domains. [file jamahealthforum-e214501-s001.pdf]

Supplemental Online Content

Srivastava T, Schmidt H, Sadecki E, Kornides ML. Disadvantage indices deployed to promote equitable allocation of COVID-19 vaccines in the US: a scoping review of differences and similarities in design. *JAMA Health Forum*. 2022;3(1):e214501. doi:10.1001/jamahealthforum.2021.4501

eMethods. Grouping of disadvantage index variables and domains under analytic meta-domains.

This supplemental material has been provided by the authors to give readers additional information about their work.

© 2022 Srivastava T et al. *JAMA Health Forum.*

eMethods. Grouping of disadvantage index variables and domains under analytic meta-domains. The nine domains shown in Figure 1 have been derived from the index-defined domains in Table 1. Most index-defined domain names were used or matched across indices, which had slight variations in domain naming. “Epidemiological Factors” were renamed as “Health.” All different “environmental” or “neighborhood”-specific factors were grouped under “Environmental and Neighborhood.” We show below the assignment of all variables and index-defined domains to the domains shown in Figure 1.

Items			Disadvantage Index			
**Domain**	**Variable**	**Index Domain**	**ADI**	**CCVI**	**HPI**	**SVI**
**Population Demographics**	**Population Estimate**					✓
	**Estimated total number of people per unit area (sq. miles)**	Population Density		✓		
	**Estimate of Daytime Population**					*
	**Persons Aged 65 +**	Epidemiological Factors (CCVI)<br><br>Household Composition & Disability (SVI)		✓		✓
	**Persons Aged < 18**	Housing type, Transportation, Household Composition & Disability (CCVI)<br><br>Household Composition & Disability (SVI)		✓		✓
	**Single-Parent Households with Children Under 18**	Housing Quality (ADI)<br><br>Housing type, Transportation, Household Composition & Disability (CCVI)<br><br>Household Composition & Disability (SVI)	✓	✓		✓
	**Percentage of family households with children under 18 with two parents**	Social			✓	
	**Percentage of registered voters**	Social			✓	

© 2022 Srivastava T et al. *JAMA Health Forum.*

	**voting in the 2012 general election**					
**Poverty**	**Persons Below Poverty**	Socioeconomic Status		✓		✓
	**Percent of families below the poverty level**	Income	✓			
	**Percent of population living below 150% of the poverty threshold**	Income	✓			
	**Percent of the population with an income exceeding 200% of federal poverty level**	Economic			✓	
**Education and Employment**	**Civilian (age 16+) Unemployed**	Socioeconomic Status (CCVI, SVI)<br><br>Employment (ADI)	✓	✓		✓
	**Percent of population aged 25-64 who are employed**	Economic			✓	
	**Percent of employed persons ≥ 16 years of age in white-collar occupations**	Employment	✓			
	**Median Family Income**	Income (ADI)<br><br>Economic (HPI)	✓		✓	
	**Per Capita Income**	Socioeconomic Status		✓		✓
	**Income Disparity**	Income	✓			
	**Percent of 3-4 year olds enrolled in pre-school**	Education			✓	
	**Percent of 15-17 year olds enrolled in school**	Education			✓	
	**Persons (age 25+) with No High School Diploma**	Socioeconomic Status		✓		✓
	**Percent of population aged ≥ 25 years with < 9 years of education**	Education	✓			
	**Percent of population aged ≥ 25 years with greater than or**	Education	✓			

© 2022 Srivastava T et al. *JAMA Health Forum.*

	equal to a high school diploma					
	Percent of population over age 25 with a bachelor's education or higher	Education			✓	
	Percentage of adults aged 18 to 64 years currently insured	Healthcare Access			✓	
	Persons without Health Insurance	Socioeconomic Status				*
	Percent of population uninsured	Socioeconomic Status		✓		
	Civilian Non-institutionalized Population with a Disability	Housing type, Transportation, Household Composition & Disability (CCVI)<br><br>Household Composition & Disability (SVI)		✓		✓
Minority Populations	Minority (all persons except white, non-Hispanic)	Minority Status & Language		✓		✓
	Index of dissimilarity	Race			✓	
	Persons (age 5+) Who Speak English "Less than Well"	Minority Status & Language		✓		✓
Housing and Transportation	Median Home Value	Housing Quality	✓			
	Median gross rent	Housing Quality	✓			
	Median monthly mortgage	Housing Quality	✓			
	Percent of low income homeowners paying more than 50% of income on housing costs	Housing			✓	
	Percent of low income renter households paying more than 50% of income on housing costs	Housing			✓	
	Percent owner-occupied housing	Housing Quality	✓		✓	

© 2022 Srivastava T et al. *JAMA Health Forum.*

	**units (home ownership rate)**					
	**Multi-Unit Structures (Housing in structures with 10+ units)**	Housing type, Transportation, Household Composition & Disability (CCVI)<br><br>Household Composition & Disability (SVI)		✓		✓
	**Mobile Homes**	Housing type, Transportation, Household Composition & Disability (CCVI)<br><br>Household Composition & Disability (SVI)		✓		✓
	**Crowding (At household level, occupied housing units, more people than rooms)**	Housing Quality (ADI)<br><br>Housing type, Transportation, Household Composition & Disability (CCVI)<br><br>Household Composition & Disability (SVI)	✓	✓		✓
	**Percent of households with less or equal to 1 occupant per room**	Housing Quality			✓	
	**Persons in Group Quarters**	Housing type, Transportation, Household Composition & Disability (CCVI)<br><br>Household Composition & Disability (SVI)		✓		✓
	**Households with No Vehicle**	Housing Quality (ADI)<br><br>Housing type, Transportation, Household Composition & Disability (CCVI)	✓	✓		✓

© 2022 Srivastava T et al. *JAMA Health Forum.*

		Household Composition & Disability (SVI)				
	**Percentage of households with access to an automobile**	Transportation			✓	
	**Percentage of workers (16 years and older) commuting by walking, cycling, or transit (excluding working from home**	Transportation			✓	
	**Percent of occupied housing units without a telephone**	Housing Quality	✓			
	**Percent of occupied housing units without complete plumbing**	Housing Quality (ADI)<br><br>Housing type, Transportation, Household Composition & Disability (CCVI)	✓	✓		
**High Risk Transmission Environments**	**Long-term care (nursing homes, assisted living, and care homes) residents per 100,000**	High Risk Environments		✓		
	**Prisons population per 100,000**	High Risk Environments		✓		
	**Percentage of population employed in high-risk industry**	High Risk Environments		✓		
**Health**	**Estimated percent of adults diagnosed with high cholesterol**	Epidemiological Factors		✓		
	**Estimated percent of adults diagnosed with a stroke**	Epidemiological Factors		✓		
	**Estimated percent of adults ever diagnosed with heart disease**	Epidemiological Factors		✓		
	**Estimated percent of adults diagnosed with chronic obstructive pulmonary disease,**	Epidemiological Factors		✓		

© 2022 Srivastava T et al. *JAMA Health Forum.*

	**emphysema, or chronic bronchitis**					
	**Estimated percent of adults reporting to smoke cigarettes**	Epidemiological Factors		✓		
	**Annual cancer incidence per 100,000 persons**	Epidemiological Factors		✓		
	**Rate of persons living with a HIV diagnosis per 100,000 people**	Epidemiological Factors		✓		
	**Estimated percent of adults reporting to be obese (a body mass index of 30 or greater)**	Epidemiological Factors		✓		
	**Estimated percent of adults ever diagnosed with diabetes**	Epidemiological Factors		✓		
**Healthcare System**	**Intensive Care Unit (ICU) Beds per 100,000**	Healthcare System Factors		✓		
	**Hospital Beds per 100,000**	Healthcare System Factors		✓		
	**Epidemiologists per 100,000**	Healthcare System Factors		✓		
	**Agency for Healthcare Research and Quality – Prevention Quality Indicator Overall Composite (PQI): admission rates for preventable conditions (via good outpatient care) adjusted per population**	Healthcare System Factors		✓		
	**Health Spending per Capita**	Healthcare System Factors		✓		
	**Aggregate cost of medical care**	Healthcare System Factors		✓		
	**Percent of population with a**	Healthcare System Factors		✓		

© 2022 Srivastava T et al. *JAMA Health Forum.*

	**Primary Care Physician**					
	**Total Public Health Emergency Preparedness (PHEP) Funding Per Capita**	Healthcare System Factors		✓		
	**Health Labs per 100,000**	Healthcare System Factors		✓		
	**Emergency Services per 100,000**	Healthcare System Factors		✓		
**Environmental and Neighborhood**	**Percent of the population living within ½ mile of a park, beach, or open space greater than 1 acre**	Neighborhood			✓	
	**Population-weighted percentage of the census tract area with tree canopy**	Neighborhood			✓	
	**Spatial distribution of gridded diesel PM emissions from on-road and non-road sources for a 2012 summer day in July (kg/day)**	Clean Environment			✓	
	**Cal EnviroScreen 3.0 drinking water contaminant index for selected contaminants**	Clean Environment			✓	
	**Mean of summer months (May-October) of the daily maximum 8-hour ozone concentration (ppm), averaged over three years (2012 to 2014)**	Clean Environment			✓	
	**Annual mean concentration of PM2.5 (average of quarterly means, µg/m3), over three years (2012 to 2014)**	Clean Environment			✓	

© 2022 Srivastava T et al. *JAMA Health Forum.*

	**Percentage of the urban and small town population residing less than ½ mile from a supermarket/large grocery store, and the percent of the rural population living less than 1 miles from a supermarket/large grocery store**	Neighborhood			✓	
	**Percentage of the population residing within ¼ mile of an off-site sales alcohol outlet**	Neighborhood			✓	
	**Combined employment density for retail, entertainment, and educational uses (jobs/acre)**	Neighborhood			✓	

*Adjunct variables in SVI 2018, excluded from SVI rankings

© 2022 Srivastava T et al. *JAMA Health Forum.*
